# Diversity and function of prevalent symbiotic marine bacteria in the genus *Endozoicomonas*

**DOI:** 10.1007/s00253-016-7777-0

**Published:** 2016-08-24

**Authors:** Matthew J. Neave, Amy Apprill, Christine Ferrier-Pagès, Christian R. Voolstra

**Affiliations:** 1 Red Sea Research Center, Division of Biological and Environmental Science and Engineering, King Abdullah University of Science and Technology (KAUST), Thuwal, 23955-6900 Saudi Arabia; 2 Woods Hole Oceanographic Institution, Woods Hole, MA USA; 3 Centre Scientifique de Monaco, 8 Quai Antoine 1er, 98000 Monaco, MC Monaco

**Keywords:** *Endozoicomonas*, Symbiosis, Marine, Coral reefs

## Abstract

*Endozoicomonas* bacteria are emerging as extremely diverse and flexible symbionts of numerous marine hosts inhabiting oceans worldwide. Their hosts range from simple invertebrate species, such as sponges and corals, to complex vertebrates, such as fish. Although widely distributed, the functional role of *Endozoicomonas* within their host microenvironment is not well understood. In this review, we provide a summary of the currently recognized hosts of *Endozoicomonas* and their global distribution. Next, the potential functional roles of *Endozoicomonas*, particularly in light of recent microscopic, genomic, and genetic analyses, are discussed. These analyses suggest that *Endozoicomonas* typically reside in aggregates within host tissues, have a free-living stage due to their large genome sizes, show signs of host and local adaptation, participate in host-associated protein and carbohydrate transport and cycling, and harbour a high degree of genomic plasticity due to the large proportion of transposable elements residing in their genomes. This review will finish with a discussion on the methodological tools currently employed to study *Endozoicomonas* and host interactions and review future avenues for studying complex host-microbial symbioses.

## Introduction

It is increasingly recognized that eukaryotic organisms rely on bacterial associates to provide a diversity of functions, from supplying nutrients and essential amino acids, to protection from pathogenic microbes and degradation of toxins (McFall-Ngai et al. 2013). Despite only being recently described (e.g. Kurahashi and Yokota 2007), the bacterial genus *Endozoicomonas* (*Gammaproteobacteria*; *Oceanospirillales*) has been reported to associate with a large diversity of marine organisms, including cnidarians, poriferans, molluscs, annelids, tunicates, and fish (Jensen et al. 2010; Morrow et al. 2012; Forget and Juniper 2013; Fiore et al. 2015; Katharios et al. 2015). They are also globally distributed and have been found living symbiotically with organisms in all major oceans of the world (Neave et al. 2016). However, although they are ubiquitously distributed, the functional role of *Endozoicomonas* is unclear. Their suggested roles have ranged from a beneficial symbiont required for healthy host functioning to a pathogen that can rapidly cause host death (Bourne et al. 2013; Katharios et al. 2015).

This mini-review provides an update on the ever-expanding range of known *Endozoicomonas* hosts and global distributions and addresses recent advancements in understanding the genetic potential and possible functions of bacteria in the genus *Endozoicomonas*. We conclude with a discussion on strategies for uncovering new insights into the lifestyle of this cryptic and enigmatic genus, including emerging tools for the study of microbial-animal symbioses, and provide recommendations for future work.

## History and prevalence of *Endozoicomonas* in the scientific literature

**Fig. 1:**
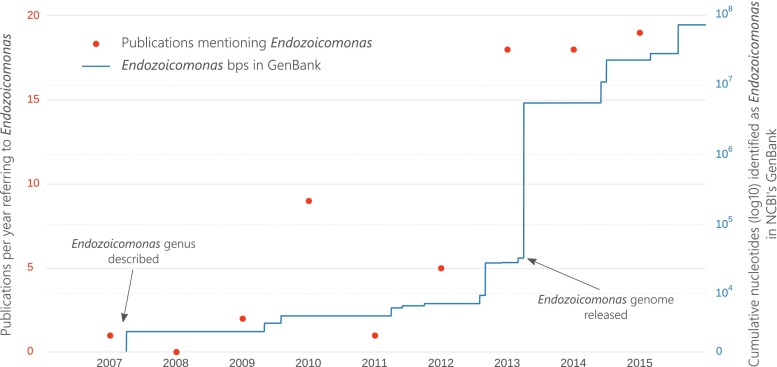
Prevalence of *Endozoicomonas* in the scientific literature as shown by the number of publications referring to the genus per year (*red markers*) and the cumulative amount of *Endozoicomonas* nucleotide information uploaded to NCBI’s GenBank (*blue line*)

The genus *Endozoicomonas* was described less than a decade ago by Kurahashi and Yokota (2007) (Fig. 1), after they isolated an unknown gammaproteobacterial symbiont from the sea slug *Elysia ornata*. Physiological and phylogenetic analyses indicated that the creation of a new genus*, Endozoicomonas* (i.e. monad living inside an animal), was required (Kurahashi and Yokota 2007). After this initial description, references to *Endozoicomonas* remained relatively scarce in the literature until 2010, when *Endozoicomonas montiporae* (Yang et al. 2010) was described, and coral symbionts with similarity to *Endozoicomonas* were discovered (Kvennefors et al. 2010). Kvennefors et al. (2010) also noted that symbionts from earlier microbiome studies (before the creation of the genus) were closely related to *Endozoicomonas*. Examples include abundant gammaproteobacterial symbionts termed “PA1” from the coral *Porites astreoides* (Rohwer et al. 2002) and *Gammaproteobacteria* from the coral *Pocillopora damicornis* (Bourne and Munn 2005). These efforts to retroactively link past studies to the *Endozoicomonas* genus, in addition to the detection of *Endozoicomonas* in new hosts, resulted in a spike in citations of *Endozoicomonas* within the scientific literature. From 2013 onwards, more than 15 publications per year referred to the genus (Fig. 1). This relatively rapid rise in *Endozoicomonas* publications, and the novelty of the genus, has led to some taxonomic inconsistencies finding their way into journal articles and databases. For example, the Greengenes database (DeSantis et al. 2006) places the genus *Endozoicomonas* in an apparently newly created family called “*Endozoicomonaceae*”. However, despite not being explicitly stated, the type description of *Endozoicomonas* contained nucleotide information that placed the genus within the family *Hahellaceae* (Kurahashi and Yokota 2007) in the order *Oceanospirillales* and this taxonomy was maintained in subsequent type descriptions (Yang et al. 2010; Nishijima et al. 2013). The incorrect term *Endozoicomonaceae* has appeared in a number of scientific publications (e.g. Dishaw et al. 2014; Katharios et al. 2015; Lawler et al. 2016) paying tribute to just how rapid this genus became scientifically popular. Confusion surrounding the initial naming of the genus has also produced inconsistencies in the scientific literature. For example, in March 2005 (prior to the *Endozoicomonas* description), a sequence named “*Spongiobacter nickelotolerans*” was submitted to GenBank (#AB205011), which is very similar (>97 % SSU rRNA identity) to the *Endozoicomonas* type strains. The associated taxonomic paper describing the isolation of *S. nickelotolerans* from a marine sponge, however, was not published and the name appears to have been abandoned (e.g. Pike et al. 2013; McCauley et al. 2016). Nevertheless, several publications have referred to “*Spongiobacter*” or to the “*Spongiobacter*/*Endozoicomonas*” group (e.g. Costa et al. 2012; La Rivière et al. 2015).

The volume of genetic data available for *Endozoicomonas* bacteria in NCBI’s GenBank (Benson et al. 2013) has also rapidly increased (Fig. 1). Since the initial deposition of the first 1437 bps SSU rRNA sequence of *E*. *elysicola* by Kurahashi and Yokota (2007), *Endozoicomonas* nucleotide information has steadily accumulated, reaching almost 100,000,000 bps by the start of 2016 (Fig. 1). Moreover, this number only takes into account data retrieved from GenBank; far more *Endozoicomonas* genetic information is available in other databases, such as NCBI’s Sequence Read Archive (SRA). Much of this rapid accumulation of genetic information can be attributed to the move towards whole genome sequencing, rather than marker gene sequencing. The first *Endozoicomonas* genome sequenced was *E*. *elysicola* in 2013 (Fig. 1) as part of the one thousand microbial genomes project (Kyrpides et al. 2014). The following year, Neave et al. (2014) released an updated version of the *E*. *elysicola* genome plus two new genomes, *E*. *montiporae* (Yang et al. 2010) and *E*. *numazuensis* (Nishijima et al. 2013). Since then, the *E*. *montiporae* genome has been re-sequenced (Ding et al. 2016), the genome of *E*. *atrinae* has been made available (Hyun et al. 2014), the genome of an undescribed pathogenic *Endozoicomonas* has been analysed (Katharios et al. 2015), and several other *Endozoicomonas* genome projects are underway (e.g. Appolinario et al. 2016). Most recently, Neave, Michell, Apprill and Voolstra (Endozoicomonas genomes reveals functional adaptation and plasticity in bacterial strains symbiotically associated with diverse marine hosts, Submitted) applied single-cell genomics and metagenomic binning to recruit four additional *Endozoicomonas* genomes from native coral host assemblages.

## Diversity and distribution of *Endozoicomonas* hosts

**Fig. 2:**
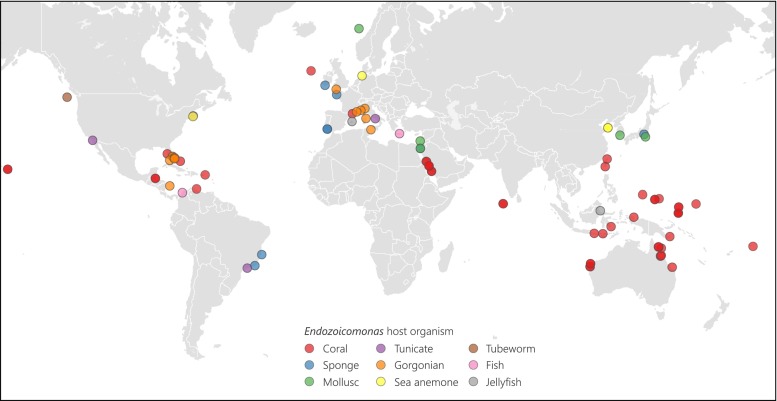
Global distribution and diversity of *Endozoicomonas* host organisms

*Endozoicomonas* symbionts have a global distribution in numerous marine hosts, from abyssal depths to warm photic zones (Fig. 2). They are most frequently detected as coral symbionts and occur throughout the global distribution of coral reefs, from the Great Barrier Reef in Australia (Bourne et al. 2008; Lema et al. 2013), Papua New Guinea (Morrow et al. 2015), Indonesia and the Pacific (Yang et al. 2010; Neave et al. 2016) to the Red Sea (Bayer et al. 2013b; Jessen et al. 2013; Neave et al. 2016; Ziegler et al. 2016), Indian Ocean (Neave et al. 2016), and the Caribbean (Morrow et al. 2012; Rodriguez-Lanetty et al. 2013) (Fig. 2). Interestingly, different coral species appear to harbour specific *Endozoicomonas* genotypes (Neave et al. 2016). For example, the sympatric corals, *Stylophora pistillata* and *Pocillopora verrucosa*, each contain different *Endozoicomonas* genotypes, and these genotypes partition differently across large geographic scales (Neave et al. 2016). Moreover, patterns of symbiont specificity seem to co-align with differences in reproductive mode. For instance, the spawning coral *P*. *verrucosa* globally associates with the same *Endozoicomonas* symbionts, whereas the brooding coral *S*. *pistillata* harbours different *Endozoicomonas* genotypes in different regions (Neave et al. 2016).

Although no quantitative methods have been applied to *Endozoicomonas*, trends in cell abundance have been inferred from SSU rRNA gene sequence abundances. These studies have linked the abundance of *Endozoicomonas* to the abundance of its coral host. For example, when the fungid coral *Ctenactis echinata* grew in its preferred Red Sea habitat, *Endozoicomonas* symbionts were more abundant than in habitats of degraded quality (Roder et al. 2015). Moreover, reduced abundances of the corals *Acropora millepora* and *Porites cylindrica* near carbon dioxide seeps in Papua New Guinea coincided with a 50 % reduction in *Endozoicomonas* symbionts (Morrow et al. 2015). Anthropogenic pollution can similarly decrease the abundance of *Endozoicomonas* bacteria. Near the large Red Sea city of Jeddah, the corals *P*. *verrucosa* and *Acropora hemprichii* contained a lower proportion of *Endozoicomonas* compared to corals further afield (Ziegler et al. 2016). In addition, bleaching of the coral *A*. *millepora* on the Great Barrier Reef induced a shift from *Endozoicomonas*-like symbionts to a *Vibrio*-dominated community (Bourne et al. 2008). Lesioned *P*. *astreoides* colonies also contained reduced *Endozoicomonas* sequence abundances, compared to non-lesioned colonies (Meyer et al. 2014). These studies suggest that *Endozoicomonas* bacteria are part of a healthy coral microbiome and reductions in their abundance may indicate unfavourable environmental conditions.

Gorgonians, commonly known as sea fans, are closely related to corals and also have a microbiome frequently dominated by *Endozoicomonas*. In the Mediterranean, the main gorgonian species (*Eunicella cavolini*, *E. singularis*, *E. verrucosa*, *Leptogorgia sarmentosa*, and *Paramuricea clavata*) harbour a microbiome dominated by *Endozoicomonas* symbionts (van de Water et al. In revision; Bayer et al. 2013a; La Rivière et al. 2015). Interestingly, patterns of host-specificity and possibly co-evolution between the gorgonian species and their specific *Endozoicomonas* genotype have also been observed (van de Water et al. In revision; La Rivière et al. 2015). In addition, another octocoral species, the red coral *Corallium rubrum*, also harbour *Endozoicomonas*, although it is much less abundant compared to the other gorgonians and is dominated by *Spirochaetales* (van de Water et al. 2016). Outside of the Mediterranean, *Endozoicomonas* symbionts were found in the gorgonian *E*. *verrucosa* living off the south-west coast of England (Ransome et al. 2014), as well as in the Caribbean gorgonian *Pseudopterogorgia elisabethae* (Correa et al. 2013). Although corals and gorgonians dominate the *Endozoicomonas* literature, these symbionts have also been found in a range of other cnidarian species. Early work by Schuett et al. (2007) detected an *Endozoicomonas* strain with 98 % similarity to *E*. *elysicola* and observed bacterial cell aggregates in the tentacles of the sea anemone *Metridium senile* from Helgoland in the North Sea. Although these aggregates were not confirmed as *Endozoicomonas*, they used scanning electron microscopy to obtain detailed images of the bacterial cells forming large aggregates within host tissues. More recently, an *Endozoicomonas* strain with 99 % similarity to *E*. *elysicola* was found in another sea anemone, *Nematostella vectensis*, from a marsh of varying salinity conditions in Massachusetts, USA (Har et al. 2015). *Endozoicomonas* symbionts also dominated two jellyfish species, *Mastigias* cf. *papua* and *Tripedalia* cf. *cystophora*, in several Indonesian lakes (Cleary et al. 2016). These examples show that *Endozoicomonas* bacteria are symbiotic with a large diversity of cnidarian species and are often abundant and host species-specific, suggesting an important and ancient evolutionary relationship with lineage-specific evolution.

*Endozoicomonas* are also known to associate with a wide range of other marine organisms (Fig. 2). A number of particularly interesting examples of the adaptability of *Endozoicomonas* come from deep-sea hydrothermal vent communities. Forget and Juniper (2013) collected the tubeworm, *Ridgeia piscesae*, from the Juan de Fuca Ridge in the north-east Pacific, which has high hydrogen sulphide concentrations (~40 μmol/L), temperatures up to 41.9 °C, and depths greater than 2000 m. Even in this extreme environment, *R*. *piscesae* contained abundant *Endozoicomonas* symbionts (Forget and Juniper 2013). Moreover, *Endozoicomonas* bacteria have been recovered from the gills of the hydrothermal vent snail *Alviniconcha* (Beinart et al. 2014), the gills of the deep-water bivalve *Acesta excavata* (Jensen et al. 2010), and from the tissues of deep-water corals (Meistertzheim et al. 2016). *Endozoicomonas* have also been associated with a number of sponge species, which are one of the oldest groups of metazoan invertebrates and often harbour a rich diversity of microbial symbionts (Rua et al. 2014). In the Caribbean, the giant barrel sponge (*Xestospongia muta*) not only contained *Endozoicomonas* bacteria, but evidence of their transcriptional activity was also detected (Fiore et al. 2015). Sponges in Brazil (Rua et al. 2014), Japan (Nishijima et al. 2013), and several other European seas also contain *Endozoicomonas* symbionts (Esteves et al. 2013; Gardères et al. 2015). In addition, tunicates, which are basal chordates, have a microbiome that consistently contains a substantial proportion of *Endozoicomonas*, suggesting that these symbionts are core members of the tunicate microbiome (Dishaw et al. 2014). Although reports to date mostly associate *Endozoicomonas* with marine invertebrates, several examples of associations with fish have also emerged in the recent literature. In these cases, fish were kept in artificial aquaculture environments and *Endozoicomonas* bacteria are suspected to have caused disease (Mendoza et al. 2013; Katharios et al. 2015). In both examples, *Endozoicomonas* formed cyst-like aggregations on the gills of the fish, resulting in epitheliocystis (Mendoza et al. 2013; Katharios et al. 2015).

The central picture that emerges is the remarkable ability of *Endozoicomonas* bacteria to adapt to a wide range of hosts and environments, from warm coral reefs to cold deep-sea mussels, and their apparent ability to transition from beneficial core microbiome members of corals and tunicates to disease-causing pathogens in fish.

## Function and genetic potential of *Endozoicomonas*

The proposed functions of *Endozoicomonas* can be summarized into three categories: nutrient acquisition and provision, structuring of the host microbiome, and roles in host health or disease (Table 1). Nutrient acquisition spans from nitrogen and carbon recycling (Nishijima et al. 2013; Forget and Juniper 2013; Correa et al. 2013; Morrow et al. 2015), or methane and sulphur cycling (Bourne et al. 2013; Forget and Juniper 2013; Correa et al. 2013; Dishaw et al. 2014; Morrow et al. 2015), to the synthesis of amino acids and other essential molecules (Neave, Michell, Apprill and Voolstra, Endozoicomonas genomes reveals functional adaptation and plasticity in bacterial strains symbiotically associated with diverse marine hosts, Submitted). Bourne et al. (2013) found that the abundance of *Endozoicomonas*-related sequences (referred to as *Oceanospirillales* sp. 1, 3, 5, and 6) in invertebrate microbial communities correlated with the presence of photosymbionts, such as *Symbiodinium* algae in coral tissues. They suggested that the photosymbionts provide carbon and sulphur to the bacteria from the large quantities of dimethylsulfopropionate (DMSP) produced (Bourne et al. 2013; Correa et al. 2013). On the other hand, *Endozoicomonas* bacteria are also found in hosts without photosymbionts (Bourne et al. 2013). In addition to nutrient cycling, *Endozoicomonas*-related members may also play a role in regulating bacterial colonization of the animal host via the production of bioactive secondary metabolites or probiotic mechanisms, such as competitive exclusion of pathogenic bacteria (Bayer et al. 2013b; Jessen et al. 2013; Rua et al. 2014; Morrow et al. 2015). Moreover, the loss of *Endozoicomonas* is often characteristic of corals with lesions, signs of disease, or if they are living in eutrophicated, warm, or acidic environments. Therefore, the abundance of *Endozoicomonas* seems to be linked with healthy colonies of diverse coral species (Morrow et al. 2012; Bayer et al. 2013b; Roder et al. 2015; Morrow et al. 2015; Ziegler et al. 2016).

**Table 1:** Suggested functions of *Endozoicomonas* bacteria

Host organism	Suggested function	Reference
Fish	Fish disease	(Mendoza et al. 2013; Katharios et al. 2015)
Sponge	Sponge health	(Gardères et al. 2015)
	Bromopyrrole production for feeding deterence	(Haber and Ilan 2014)
	Carbohydrate fermentation/nitrate reduction	(Nishijima et al. 2013)
	Antibiotic production	(Rua et al. 2014)
Tunicate	Sulphur cycling/nutrient metabolism	(Dishaw et al. 2014)
Hydrothermal vent snail	Host nutrition/sulphur cycling or breakdown of organic compounds	(Beinart et al. 2014)
Hydrothermal polychaete	Methane cycling/food degradation	(Forget and Juniper 2013)
Scleractinian corals	Quorum-sensing molecules	(Bayer et al. 2013b)
	Microbiome structuring	(Jessen et al. 2013)
	Antimicrobial activity/N-acyl homoserine lactones	(Morrow et al. 2015)
	Coral health	(Meyer et al. 2014; Roder et al. 2015; Webster et al. 2016)
	Coral health and/or disease	(Ziegler et al. 2016)
	Protection from bleaching	(Pantos et al. 2015)
	Dimethylsulfoniopropionate (DMSP) metabolism/sulphur cycling	(Raina et al. 2009; Bourne et al. 2013; Correa et al. 2013)
	Carbohydrate metabolism/nutrient acquisition	(Correa et al. 2013; Morrow et al. 2015)
Octocoral (gorgonians)	Host health	(Vezzulli et al. 2013; Ransome et al. 2014)

**Fig. 3:**
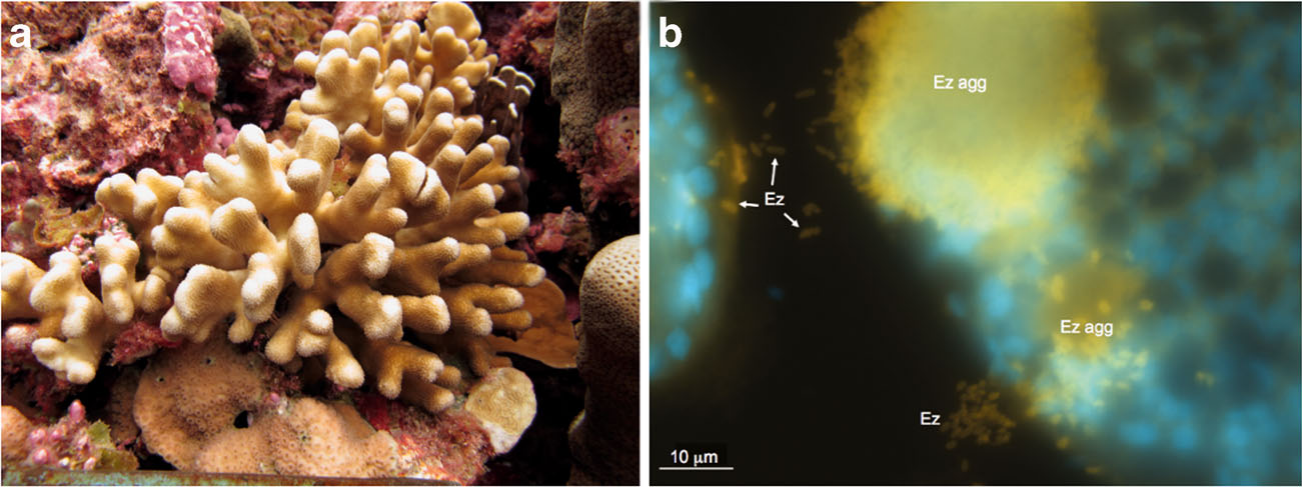
Photograph of *S*. *pistillata* colony from Nukuoro atoll, Federated States of Micronesia (**a**), and microscopic photo of *Endozoicomonas* (*Ez*) probed cells (*yellow*) within the tentacles of *S. pistillata* residing in aggregates (*Ez agg*) as well as just outside the aggregate (**b**). *Endozoicomonas* cells are hybridized with the horseradish peroxidase-labelled probe targeting the *Endozoicomonas* bacteria, and the blue staining is a general nucleic acid stain (DAPI) (methodology detailed in Neave et al. (2016))

The spatial location of *Endozoicomonas* bacteria within host tissues may also have functional implications. For example, *Endozoicomonas* frequently form aggregations in various host habitats (Bayer et al. 2013b; Mendoza et al. 2013; Katharios et al. 2015; Schreiber et al. 2016). In corals, fluorescent oligonucleotides have been designed for *Endozoicomonas* and used to hybridize to *Endozoicomonas* cells, confirming their residence within host tissues (Bayer et al. 2013b), where they formed similar structures to cell-associated microbial aggregates or CAMAs as previously described using histological staining in corals (Work and Aeby 2014). More recently, using catalyzed reporter deposition fluorescence in situ hybridization (CARD-FISH) with this same probe, bacterial cells have been better resolved within the autofluorescent coral tissues and it was found that *Endozoicomonas* cells form dense aggregations and can reside within the tentacles of corals (Fig. 3; Neave et al. 2016). Aggregations filled with thousands of bacterial cells have also been found in anemone tentacles, and although SSU rRNA gene sequence data demonstrates that *Endozoicomonas* are in residence with these anemones, the tentacle-associated cells have not yet been confirmed as *Endozoicomonas* (Schuett et al. 2007). Another probe developed for *Endozoicomonas* also identified cells residing in extracellular aggregations in sea squirts (Schreiber et al. 2016). In fish, *Endozoicomonas* also form extremely dense aggregations containing thousands of individuals surrounded by a thin tightly enveloping membrane (Mendoza et al. 2013; Katharios et al. 2015). These formations, particularly the membrane barrier, may provide protection from host immune cells or other host responses to bacterial infection. Functionally, the aggregations may act as centres of protein transformation and production that could be beneficial for the host. Moreover, several genotypes of *Endozoicomonas* are known to inhabit individual hosts (Neave et al. 2016), and the aggregations could be comprised of several complementary genotypes, or even different bacterial species, that work together to passage nutrients and proteins. The formation of aggregations also suggests that some form of cell-to-cell communication is required, such as quorum-sensing molecules (Waters and Bassler 2005). To date, all *Endozoicomonas* microscopy studies have found aggregations in host tissues, suggesting that these formations are an important part of *Endozoicomonas* function and colonization.

The recent availability of *Endozoicomonas* whole genome sequences has significantly improved our understanding of their genetic potential, possible functional roles, and life cycle. These data have revealed that *Endozoicomonas* genomes are relatively large, ranging from 5.60 Mbs for *E*. *montiporae* (Neave et al. 2014) to 6.83 Mbs for *E*. *atrinae* (Hyun et al. 2014). The genomes support a correspondingly large number of protein-coding genes, suggesting that genome streamlining, which often occurs in symbiotic bacteria (e.g. Kwan et al. 2012), is not occurring or at least not predominantly. This may indicate that *Endozoicomonas* have periods during their lifecycle that require a full complement of genetic pathways, e.g. a free-living stage. In a recent study of the *E*. *montiporae* genome, Ding et al. (2016) found genes coding for an *N-*deglycosylation enzyme that was hypothesized to help with penetration of the mucus layer of their coral host. They also found genes that may be involved in initiating internalization and evasion of the host immune response and hypothesized that *Endozoicomonas* was a beneficial symbiont that could help increase the efficiency of host gluconeogenesis (Ding et al. 2016). Interestingly, a high proportion of repeat sequences was found in the *E*. *montiporae* genome (Ding et al. 2016), which is similar to what was reported for a pathogenic *Endozoicomonas* strain (Katharios et al. 2015). This suggests that repeat and insertion sequences may help *Endozoicomonas* strains to adapt to new hosts or to transition between mutualistic and parasitic lifestyles. Importantly, Neave, Michell, Apprill and Voolstra (Endozoicomonas genomes reveals functional adaptation and plasticity in bacterial strains symbiotically associated with diverse marine hosts, Submitted) comparatively analysed the genomes of *E*. *elysicola*, *E*. *montiporae*, *E*. *numazuensis*, and four newly sequenced *Endozoicomonas* strains from the Red Sea corals *S*. *pistillata*, *P*. *verrucosa*, and *Acropora humilis* and found a high proportion of transposable elements in the *Endozoicomonas* genomes, further implying that *Endozoicomonas* use these elements to rapidly evolve to new hosts or niches. In addition, the *Endozoicomonas* genomes were enriched for carbon sugar transport and protein secretion, suggesting that they contribute to carbohydrate cycling and delivery to their host organism (Neave, Michell, Apprill and Voolstra, Endozoicomonas genomes reveals functional adaptation and plasticity in bacterial strains symbiotically associated with diverse marine hosts, Submitted). The common denominator among *Endozoicomonas* genome projects is the high incidence of transposable elements incorporated into their genomes, possibly allowing for rapid adaptation. These genomic findings are corroborated by field evidence showing that *Endozoicomonas* are associated with numerous hosts in numerous marine habitats. The genomes also tend to show the ability to transport and transform protein products that may then be used by the host. Although these studies provide important advances, future studies may be improved by obtaining in situ gene expression data or other forms of functional information, in order to refine our understanding of the specific role of *Endozoicomonas* with a particular host.

## Future prospects—novel techniques to examine microbial functions

The functional role of *Endozoicomonas* bacteria within their many hosts is poorly understood. One fundamental but unknown aspect of many *Endozoicomonas* species is their genomic repertoire and metabolic potential. This question has traditionally been difficult to answer; however, new and emerging methods for determining bacterial function are likely to provide fascinating insights. Currently, many techniques that aim to discover bacterial function rely on the availability of cultured isolates. For example, bacterial cultures allow for efficient whole genome sequencing, for infectivity trials, and for traditional bacteriological tests of physiological and nutritional requirements. Although a number of *Endozoicomonas* species have been successfully cultured, many species are not readily amenable to isolation from host tissues (e.g. Katharios et al. 2015). In these cases, culture-independent methods can be used to obtain valuable data, such as whole genome sequences. For example, Katharios et al. (2015) micro-manipulated *Endozoicomonas* aggregates from sections of fish tissue, sequenced the DNA using high throughput technologies, and obtained a novel draft genome that provided valuable insights. Moreover, in cases where *Endozoicomonas* aggregates are not easily micro-manipulated, methods such as metagenomic binning and single-cell genomics can be used to acquire whole genome sequences. Recently, the genomes of *Endozoicomonas* associated with different species of Red Sea corals were obtained by differential coverage binning of coral metagenomes, as outlined by Albertsen et al. (2013), and by sequencing single-cell isolates (Neave, Michell, Apprill and Voolstra, Endozoicomonas genomes reveals functional adaptation and plasticity in bacterial strains symbiotically associated with diverse marine hosts, Submitted). With further improvements in metagenomic binning software (e.g. Imelfort et al. 2014; Alneberg et al. 2014; Eren et al. 2015) and in single-cell genomic methods, these may become useful for rapidly obtaining multiple genomes at a relatively small cost. A major improvement to these pipelines would be the addition of techniques that obtain gene expression data. One possible avenue that is already being developed is the use of RNA-Seq on isolated single cells (e.g. Wang and Navin 2015). Another is RNA subtraction technology, where total RNA is collected from host tissues, ribosomal RNA and eukaryotic messenger RNA are removed, and the remaining bacterial mRNA is sequenced (e.g. Stewart et al. 2010; Daniels et al. 2015). When combined, these techniques could provide fascinating insights into the intricate associations of *Endozoicomonas*-animal symbioses.

Although whole genome sequences and gene expression data are valuable, they cannot completely uncover the metabolic and physiological dynamics of *Endozoicomonas* bacteria in situ. For example, questions of how *Endozoicomonas* aggregates share metabolites with the host, or which *Endozoicomonas* genotypes are represented in aggregates, are difficult to answer. These questions may become tractable with the development of secondary ion mass spectrometry (SIMS), which has opened a new frontier in microbial ecology. While previously measurements of bacterial metabolism and physiology were restricted to community-based culture or whole-animal measurements, these novel technologies allow for the quantification of single-cell metabolisms at the spatial resolution of electron microscopy (Pernice and Levy 2014). Thereby, these technologies not only enable one to localize and quantify metabolic activity of selected tissues or microorganisms, but can also account for single-cell heterogeneity, a previously overlooked phenomenon, and of particular importance when hosts harbour multiple strains of *Endozoicomonas* (e.g. Neave et al. 2016). There are several SIMS technologies used to investigate metabolic functioning of bacteria in culture or within a host-symbiont framework. Nanoscale SIMS (NanoSIMS) is used to quantify stable isotope ratios at high spatial resolution. Coupled with pulse-chase isotope labelling, this technology can be used to detect the uptake, incorporation, and transfer of metabolites (Kopp et al. 2013). This technique may be particularly useful in detecting molecules that are consumed and produced by *Endozoicomonas* aggregations, providing data on molecule transformation within aggregations and allowing fascinating insights into potential benefits for the host. A recently developed modification of the traditional NanoSIMS approach is halogen in situ hybridization SIMS (HISH-SIMS) (Alonso et al. 2012), where specific probes are used to label targeted cells, which allows identification of the precise location of microbes within host tissue. This modification would permit *Endozoicomonas* aggregations to be interrogated for single-cell heterogeneity, including different genotypes or different bacterial species, and assess the relative contributions of these to aggregate functioning. Another technique called Time-of-Flight SIMS (ToF-SIMS) uses high-resolution mass spectrometry to identify molecules within a given cell or tissue (Colliver et al. 1997). This technology can be used to identify metabolic processes within microorganisms or the compounds they release, which would provide metabolic data at the level of individual *Endozoicomonas* cells and genotypes. While all of these technologies have proven to be powerful tools in understanding microbial function, they are even more powerful when combined. For instance, using Nano-, HISH- and ToF-SIMS on the same biological sample would make it possible to highlight specific *Endozoicomonas* genotypes in a host organism background and to characterize and quantify the nature of translocated metabolites, which should provide quantum leaps in our understanding of the function of host-associated *Endozoicomonas* bacteria.

## Conclusions and outlook

*Endozoicomonas* bacteria are rapidly being recognized as globally important marine symbionts. Few other bacterial genera are symbiotic with such a large diversity and distribution of host species, suggesting that the function and adaptability of *Endozoicomonas* bacteria are particularly exceptional. Indeed, several *Endozoicomonas* species have unusually flexible genomes and a great diversity of metabolic pathways. Studies on the exact functional roles of *Endozoicomonas* within their many hosts are only in their infancy, and many exciting and novel discoveries await. Moreover, with new technologies allowing for in situ genome sequencing, gene expression analysis, and the measurement of metabolites, future functional discoveries will be greatly accelerated.
